# Vasculitis–panniculitis mimicking unresolved diabetic foot ulcer

**DOI:** 10.1530/EDM-22-0397

**Published:** 2024-01-08

**Authors:** Debby Christiana Soemitha, Deshinta Putri Mulya, Hemi Sinorita

**Affiliations:** 1 Department of Internal Medicine, Faculty of Medicine, Universitas Gadjah Mada, Yogyakarta, Indonesia; 2 Division of Allergy and Immunology, Department of Internal Medicine, Faculty of Medicine, Universitas Gadjah Mada, Yogyakarta, Indonesia; 3 Division of Endocrinology, Metabolism and Diabetes, Department of Internal Medicine, Faculty of Medicine, Universitas Gadjah Mada, Yogyakarta, Indonesia

**Keywords:** Adult, Male, Asian - other, Indonesia, Skin, Diabetes, Cardiovascular endocrinology, Unique/unexpected symptoms or presentations of a disease, January, 2024

## Abstract

**Summary:**

Diabetes foot ulcer (DFU) is a common long-term complication of diabetes. Intractable chronic wounds to standard care of diabetic foot raise the question of whether other factors intervene in disease development. We report a case of a 54-year-old male patient who came to Sardjito General Hospital with leg pain and previous history of multiple debridement and amputation for DFU referred from a remote hospital yet no improvement was evident in the surrounding lesion following treatment. Consequently, a histopathological examination was carried out proving the presence of other aetiologic factors, vasculitis and panniculitis existing in the lesion. In this case, we report a rare type of causative factor of foot ulcers among diabetic patients. Vasculitis suspected for polyarteritis nodosa accompanied by panniculitis is considered in this patient. The treatment of choice is corticosteroids or immunosuppressants based on the clinical condition, contrary to usual wound care in DFU. Based on the evidence, clinicians need to consider other causes than only macrovascular complications in a diabetic patient with DFU that is intractable to standard wound care. In this patient, vasculitis may be considered in forming diabetic foot ulcers alongside macrovascular complications.

**Learning points:**

- A thorough examination is essential to rule out other processes in intractable DFU patients.
- Prompt management based on proper diagnosis is crucial to prevent peripheral arterial disease complications.
- Vasculitis and macrovascular complication are inseparable processes forming DFU in this patient.

## Background

Diabetes mellitus (DM) is a chronic metabolic disease associated with vascular pathology, commonly leading to peripheral arterial disease (PAD). Based on International Diabetes Federation, Indonesia ranks sixth globally for diabetic prevalence. About 67.9% of the diabetic population in Indonesia fails to achieve the targeted HbA1c level (<7%), making macrovascular complications a common incidence in this population. A diabetic foot ulcer (DFU) is another frequent diabetes complication led by the combination of poor glycaemic control, neuropathy, infection, and PAD. DFU is localised in the pressurised area with repetitive trauma, commonly intractable and chronic in nature. This clinical entity emerges as a complex complication in type 2 DM involving the partnership among podiatrist, endocrinologist, vascular specialist, and infectious disease expert for excellent outcomes. DFU is estimated at 7.3% among type 2 DM patients reported in Indonesia (1). Notwithstanding its linear aetiology with diabetes, the evaluation of DFU patients for exploring other causes is justifiable, which are recalcitrant to standard DFU wound care management.

As DFUs fall under the classification of chronic wounds, it becomes evident that chronic wounds encompass wounds with a delayed wound-healing process (2). These types of wounds emerge not only as a substantial economic burden but also inflict psychological distress upon patients and their families. There are established red lines between neuro-ischaemic and neuropathic factors elucidated in the pathogenesis of DFUs (3, 4). However, a comprehensive investigation of underlying causes for delayed wound healing is essential. Vasculitis and autoimmune wounds also present as chronic wounds; it is estimated that more than 20% of intractable wounds are associated with other aetiologies, such as vasculitis, pyoderma gangrenosum and other autoimmune diseases (5). Through this case report, We demonstrate the evidence of another causative factor associated with the forming of DFUs in a DM Type 2 patient that is intractable to the standard wound care and vascular intervention. A shifting therapeutic approach using corticosteroids or immunosuppressants is indispensable following an autoimmune process of vasculitis and panniculitis confirmed, although this is opposed to the usual standard diabetic wound care.

## Case presentation

A 54-year-old male patient was referred to Sardjito General Hospital, a tertiary health centre in Yogyakarta, Indonesia. He presented with a chief complaint of persistent left leg pain in the left leg after serial amputation procedures carried out at a secondary healthcare facility over the preceding 2 months. Through anamnesis, the initial manifestation was a blister-like lesion on the sole of the left foot with 2 cm in diameter. Due to its progression, the patient sought medical attention, leading to the decision to perform necessary debridement. Following 1 month, the wound did not show any significant improvement prompting the patient to undergo a second debridement. The same complaints were recurring, and subsequent postoperative evaluation revealed a deteriorating condition rather than wound resolution, with newly gangrenous lesions appearing in other locations, notably at the tip of the left toe.

**Figure 1:**
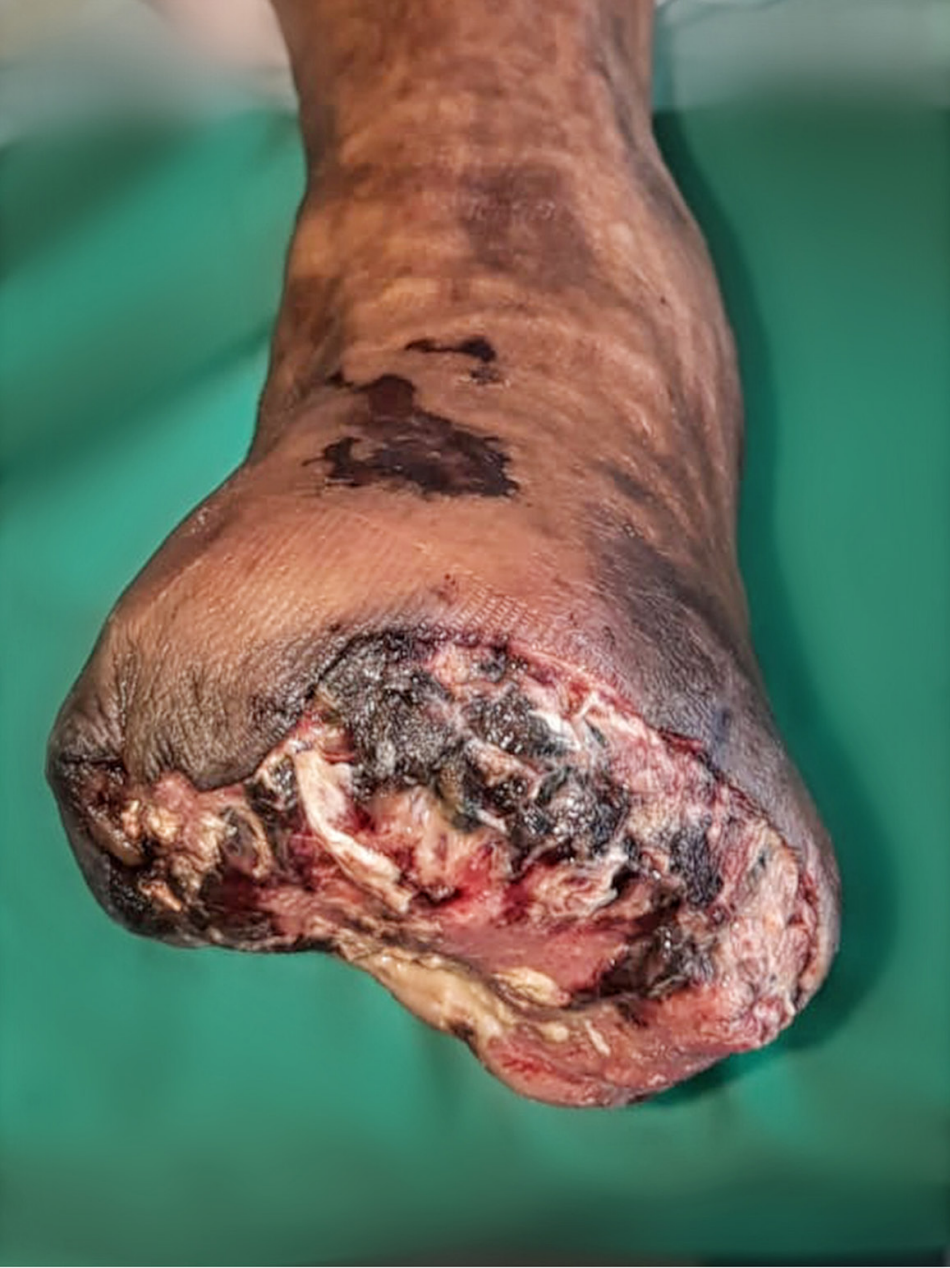
Chronic wounds after first debridement and amputation of digits I–V of the left foot.

In the emergency room, oxygen saturation assessments on the right–left (R/L) toe yielded the following results: digit 1 – R: 96%, L: 85%; digit II – R: 99%, L: 79%; digit III – R: 95%, L: 84%; digit IV – R: 96%, L: 70%; digit V – R: 99%, L: 42%. Arteriography and Doppler ultrasound were performed revealing significant stenotic lesion spanning the proximal–distal regions of the left anterior tibialis artery and the distal left peroneus artery. Based on clinical examination, imaging study, and arteriography, a Lisfranc amputation involving digits II–V of the left foot was undertaken. However, the postoperative wound remained shrouded in marked inflammation, defying resolution even after employing standard wound care and multiple surgical interventions (Fig. 1).

## Investigation

The patient’s persistent complaints range from pain and postoperative wound issue following Lisfranc amputation in the hospital, notwithstanding the appropriateness of the procedure based on imaging studies. Postoperatively, pus accumulation was notable on the surgical site, coupled with visible necrotic tissue; additionally, the first digit of the left foot demonstrated a darkish skin discolouration, representing a new process of ischaemic injury. Consequently, a subsequent debridement and second amputation of the left digit I were tabled. At this point, it becomes imperative to solicit alternative causal factors for two reasons. First, notwithstanding glycaemic control, infection remains a likely contributor to recent clinical conditions. However, the infection process was under control through antibiotic administration, as per a de-escalation strategy. In addition, the wound appearance with a darkish spot in the distal part of the foot represented more a vascular lesion rather infective process. Secondly, consideration of an autoimmune process, specifically vasculitis, holds merit. Through follow-up in the hospital, there was a bilateral distribution of lesions (black spots), that emerge several days during hospital admission concurrent with DFU of the left foot.

**Figure 2:**
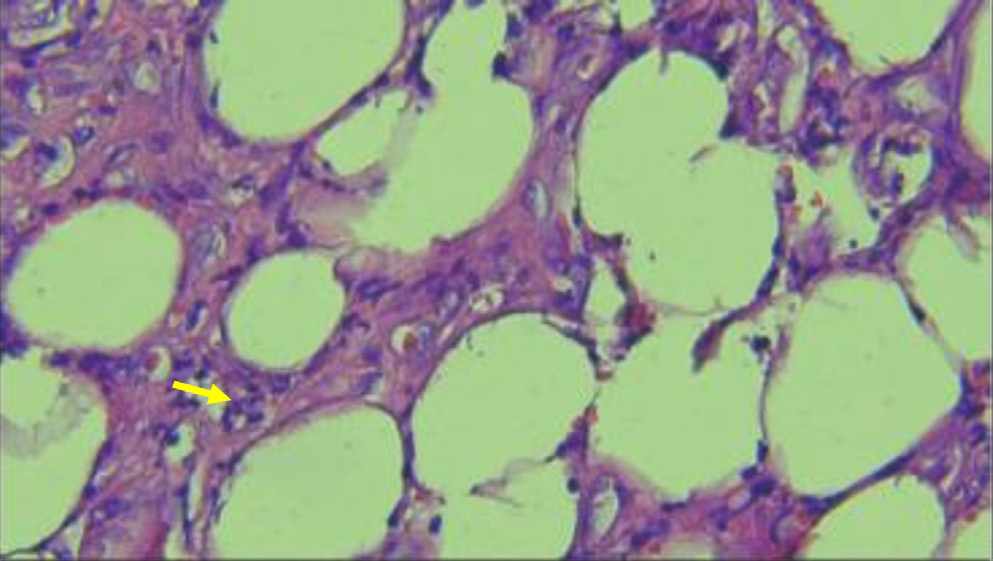
Histopathological examination showed panniculitis (yellow arrow).

**Figure 3:**
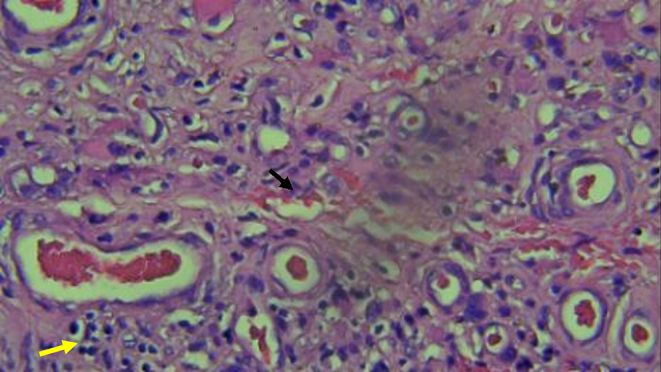
Histopathological examination demonstrated vasculitis (black arrow).

To prove the suspicion of vasculitis, a histopathological examination was undertaken between the healthy and necrotic regions in both lower limbs. Histopathology expertise was characterised by the presence of blood vessel proliferation and infiltration of inflammatory cells, such as lymphocytes, plasma cells and neutrophils, similar findings were observed in the dermis as well as the subcutaneous layer accompanied by a large area of necrosis and fibrinoid necrosis in involved blood vessels. PMN infiltration was observed inside the amputated region whereas normal findings were found in healthy tissue (Fig. 2 and 3). Histopathology concluded that post-debridement tissue showed extensive necrosis and granulation tissue in suppurative inflammation accompanied by vasculitis and panniculitis.

## Treatment

Based on histopathological examination, the diagnosis of vasculitis and panniculitis was evident. Four main strategies were considered necessary in this patient: controlling inflammation by administering steroid or oral immunosuppressants; infection control; anti-platelet agents to prevent re-stenosis; and blood glucose control to maintain within the target below 140 mg/dL. Initially, a steroid was used to control inflammation, methylprednisolone 1 × 16 mg for 2 weeks and then the dose was tapered to 8 mg once daily, but when the blood glucose became unpredictable following adjusting insulin dosage, this medication was switched to azathioprine 1 mg/kg BW. For infection prevention, a wound dressing change every 2 days was performed in addition to weekly monitoring in the diabetic foot outpatient clinic by the respected podiatrist. Since imaging studies proved the evidence of PAD, long-term use of aspirin for this patient was administered. The patient was administered insulin (dosage: 0.3–0.4 U/kg/day) and blood glucose ranged from 150 to 200 mg/dL.

## Outcome and follow-up

**Figure 4:**
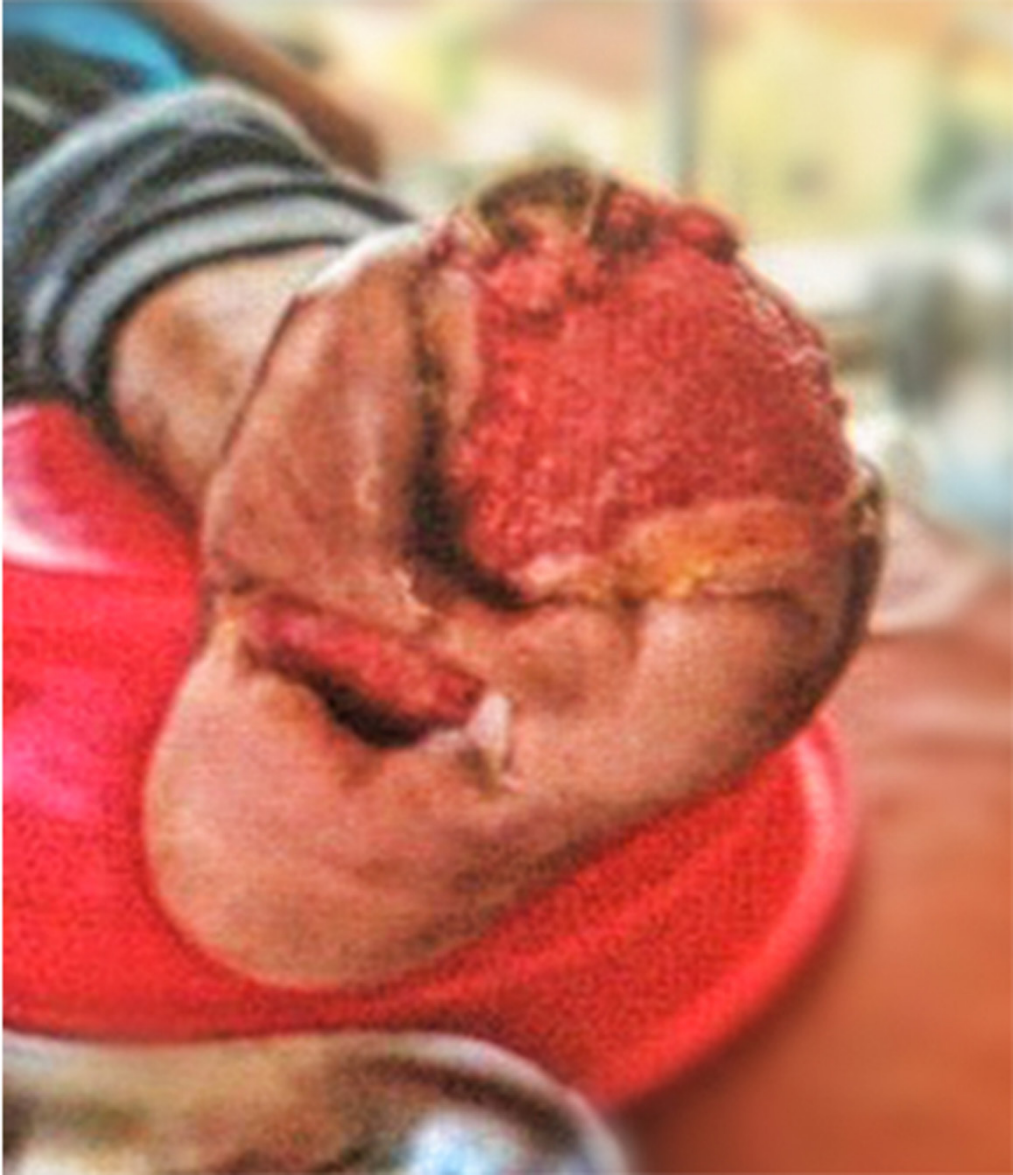
Patient’s wound development following 2 months of amputation and debridement as well as corticosteroid therapy.

The patient was declared pain-free following the administration of corticosteroids and subsequent therapy of oral immunosuppressant azathioprine. Leg wounds improved progressively without any marked inflammation. Following several weeks of steroid and immunosuppressant, the necrotic lesion had vanished with significant granulation tissue appearing surrounding post-amputation wounds (Fig. 4). Patient’s quality of life has been improved and he is able to continue his daily activities.

## Discussion

Consideration of vasculitis or other autoimmune diseases should be considered when confronted with chronic wounds that demonstrate recalcitrant against both vascular interventions and standard wound care protocols. While their proportion is lower compared to ordinary DFU, vasculitis and autoimmune diseases are more likely to manifest in cases of chronic lower extremity ulcers that simulate unresolved macrovascular complications of DM. Notably, a subset of poorly healing wounds with a prevalence of 20–23% that remain unresponsive to vascular intervention can be attributed to alternative causes, including vasculitis, rheumatoid arthritis, systemic lupus erythematosus, scleroderma, pyoderma gangrenosum, and other autoimmune diseases (5, 6).

Systemic vasculitis is an inflammatory of the blood vessels, categorised by the size of the affected blood vessels – encompassing large, medium, and small blood vessels. Concurrently, panniculitis is a disorder characterised by inflammation, induration, and ulceration of adipose and subcutaneous tissue; it may co-occur with nodular vasculitis, erythema nodosum, or erythema induratum. Alternatively, panniculitis might stem from pancreatic disorders or α1-antitrypsin deficiency (5). Meanwhile, the histopathological classification of panniculitis ranges of several subdivisions, including septal panniculitis devoid of vasculitis, septal panniculitis with vasculitis, lobular panniculitis devoid of vasculitis, and lobular panniculitis accompanied by vasculitis (7). The histological classification of panniculitis correlates with the size of the afflicted vessels, with the majority of septal lesions affecting larger to medium-sized vessels, while most lobular lesions are observed in smaller vessels.

In the context of the presented case, the lesion was identified predominantly in medium-sized blood vessels, coinciding with vasculitis. Consequently, the panniculitis subtype refers to septal lesions that primarily involve arteries. This presentation suggests that cutaneous polyarthritis nodosa (CPAN) might be the cause of the observed lesions. The potential transformation of CPAN confined to the skin into polyarteritis nodosa (PAN) remains a subject of ongoing debate. CPAN is observed in 25–60% of PAN cases and can also present alongside extracutaneous manifestations, including peripheral neuropathy and myalgia. Following the diagnostic criteria for PAN, the presence of a skin lesion plus at least one of the extracutaneous manifestations is sufficient for confirming the diagnosis of PAN (8).

Based on the American College of Rheumatology Foundation Guideline 2021, the mainstay treatment for vasculitis and panniculitis lies in the utilisation of corticosteroids and immunosuppressants. Steroid or immunosuppressant dosing is based on disease status (active, severe, non-severe, remission, refractory, and relapse) (9). For patients with a prior diabetic history, it should be noted that administering corticosteroids can temporarily increase blood sugar levels. Consequently, tight blood sugar control is necessary to optimise wound healing outcomes. Based on our experience, this case report is the first instance of a patient diagnosed with a combination of vasculitis and panniculitis producing PAD that further complicated DFU natural history in a diabetic individual. The intricate interplay between the pathogenesis of diabetes and chronic vascular inflammation underscores the inseparable clinical entity, rendering their simultaneous management of the two conditions critical.

## Declaration of interest

The authors declare that there is no conflict of interest that could be perceived as prejudicing the impartiality of the case study reported.

## Funding

This case report did not receive any specific grant from any funding agency in the public, commercial, or not-for-profit sector.

## Patient consent

The patient has given written informed consent for the publication of the case report.

## Author contribution statement

All authors made equal contributions to the preparation of the manuscript.
